# Can the Dynamic External Pelvimetry Test in Late Pregnancy Reveal Obstructed and Prolonged Labor? Results From a Pilot Study

**DOI:** 10.7759/cureus.20566

**Published:** 2021-12-21

**Authors:** Marco Siccardi, Cristina Valle

**Affiliations:** 1 Obstetrics and Gynecology, Primal Osteopathy Institute, Savona, ITA; 2 Obstetrics and Gynecology, San Paolo Hospital, Savona, ITA; 3 Yoga and Cranial Osteopathy, Primal Osteopathy Institute, Savona, ITA

**Keywords:** biomechanics, dystocia, pelvimetry, childbirth, pregnancy, operative delivery, fetal-pelvic disproportion, pelvis

## Abstract

Background

The size and mobility of the maternal pelvic space are fundamental factors in successful childbirth and can allow operators to screen for dystocia. This pilot study including a group of 70 pregnant women aimed to test whether the external dynamic pelvimetry test can be used to predict the likelihood of obstructed labor.

Methodology

The study cohort consisted of 70 pregnant women in their third trimester. The cohort was divided retrospectively into an obstructed labor group and a control group. Obstructed labor was defined using the following obstetric outcomes: augmentation with oxytocin from the first phase of the dilating period, Kristeller’s maneuvers, vacuum extractor (kiwi), forceps, and the cesarean section following the onset of labor.

Results

The measurements obtained for the longitudinal hemi-diameter of Michaelis, the inter-tuberous diameter, and the base of the Trillat’s triangle were statistically significant in every position. The difference in the measurements of the transverse diameter of Michaelis between standing and hands-and-knees position and the difference in the sizes of the bi-cristal diameter between hands-and-knees and squatting position were statistically significant.

Conclusions

Dimension and biomechanical properties of the pelvic tissue and spaces influence the evolutionary childbirth process. After clinical confirmation on a large population, hypomobility of specified external pelvic diameters measured in shifting positions can become a screening tool to detect the contracted pelvis and prevent damage caused by dystocia and prolonged labor in women and newborns.

## Introduction

Cephalopelvic disproportion (CPD) is a common indication of cesarean section in labor and is believed to be the leading cause of obstructed and prolonged labor as well as instrumental delivery [1]. If dystocia in labor is not diagnosed on time, it can lead to maternal and neonatal complications. CPD can lead to severe complications for both the mother and the baby if not managed promptly. Obstructed labor, the direct clinical consequence of disproportion, is responsible for approximately 8% of maternal deaths worldwide [2]. In exploited and low-resource countries with a low possibility of medical assistance, socially relevant complications include postpartum hemorrhage, postpartum infections, genital fistulas, pelvic floor dysfunction, uterine rupture, and maternal death. Fetal and neonatal complications such as asphyxia, septicemia, damage to the central nervous system, and death may also occur [2].

CPD is a typical indication for medically assisted birth in modern societies, increasing the rate of cesarean deliveries worldwide, but a true CPD is a rare event [1]. Therefore, the large size of the fetal head may not be a unique or even the primary cause of obstructed labor. Consequently, it is important to consider the dimensions and plasticity of the maternal bony canal as essential in the diagnostic evaluation in obstetrics practice to prevent dystocia. In the remote and recent past, internal and external pelvimetry (clinical, radiological, and anthropometric reconstructions) has been utilized to evaluate the maternal pelvis adequately [3-7]. However, the research findings are inconclusive due to a mix of promising and disappointing studies for diagnosing and screening the “contracted pelvis” in traditional obstetrics.

Interestingly, research with high-tech instruments (magnetic resonance, optoelectronic devices, and three-dimensional computed reconstruction) has demonstrated the possibility of measuring the maternal pelvis in different positions, affirming the change in the measurements of the pelvic diameters. Changing pelvic diameter with postures shows the dynamic plasticity of maternal tissues that may occur and influence the course of childbirth [3-8].

Pregnancy, delivery, and birth are dynamic processes. If uncomplicated, childbirth can be performed safely at home, in a non-hospital facility, or in a low-resource setting. It is assisted in most countries by midwives without medical assistance, and pregnant women are encouraged to assume different positions for facilitating labor. Changing posture makes the descent of the presenting part easier and decreases labor complications [8,9]. Pelvic hypomobility and connective tissue stiffness can cause pain and negatively affect labor and delivery. Obstructed and prolonged labor is unsafe for the mother and the child to manage outside the hospital environment [1,2]. Recently, studies have again approached the dimensions of the sacral area of Michaelis and some other diameters used in traditional external pelvimetry, highlighting a possible utility in detecting the need for operative deliveries caused by CPD before labor onset [10-14]. Our previous studies showed the ability of external obstetric pelvimetry to measure the maternal diameters in different positions. We found that as the woman changed her posture, the diameters varied, affirming the findings of studies that used high-tech methods to obtain these measurements [15,16].

The present pilot study is a part of the research concerning dynamic pelvimetry. It aims to test the value of assessing the dynamic external pelvimetry (DEP) test performed in the late pregnancy period to detect the risk of labor augmentation and operative delivery before labor onset. To our knowledge, no study has yet evaluated the feasibility of pre-screening for obstructed and prolonged labor using pelvic diameter measurements obtained in different maternal positions using this low-tech method of external pelvimetry.

## Materials and methods

The present pilot study is a longitudinal and cross-sectional, observational case-control research, following a previous report published in the Cureus Journal of Medical Science [15]. It is a part of the “Osteopaths in Obstetrics” study and updating program (Institutional Review Board of San Paolo Hospital issued approval number 2019/119933), which aims to deepen the relationship between osteopathy, yoga in pregnancy, and pelvic mobility. The Technical/Administrative Secretariat of the Ethics Committee of the ASL2 was responsible for registering the main study with the Regional Ethics Committee. The analysis was performed at the Obstetrics Department of San Paolo Hospital in Savona, Italy. The study followed the Strengthening the Reporting of Observational Studies in Epidemiology (STROBE) statement checklist and was conducted in conformity with the Helsinki Declaration.

After obtaining informed consent, the first author performed the DEP test on pregnant women in their last trimester received at the “practice for physiological pregnancy” of the Obstetrics Department between January 10 and October 30, 2020. Data were registered on the clinical records of the pregnant women, and the labor and delivery data were obtained from the clinical records after dismissal from the hospital. For this study, we selected 70 patient records with complete DEP tests and labor and delivery data among low-risk pregnancies. Low-risk pregnancies were certified using medical records. High-risk pregnancies, planned cesarean sections, premature births, and twin pregnancies were excluded. Only individuals who did not report abdominal, pelvic, or vertebral surgical interventions, did not suffer from lumbar-pelvic pains, did not have previous obstetrics complications, and did not have abdominal-pelvic diseases at the clinical evaluation were included in the study.

**Figure 1:**
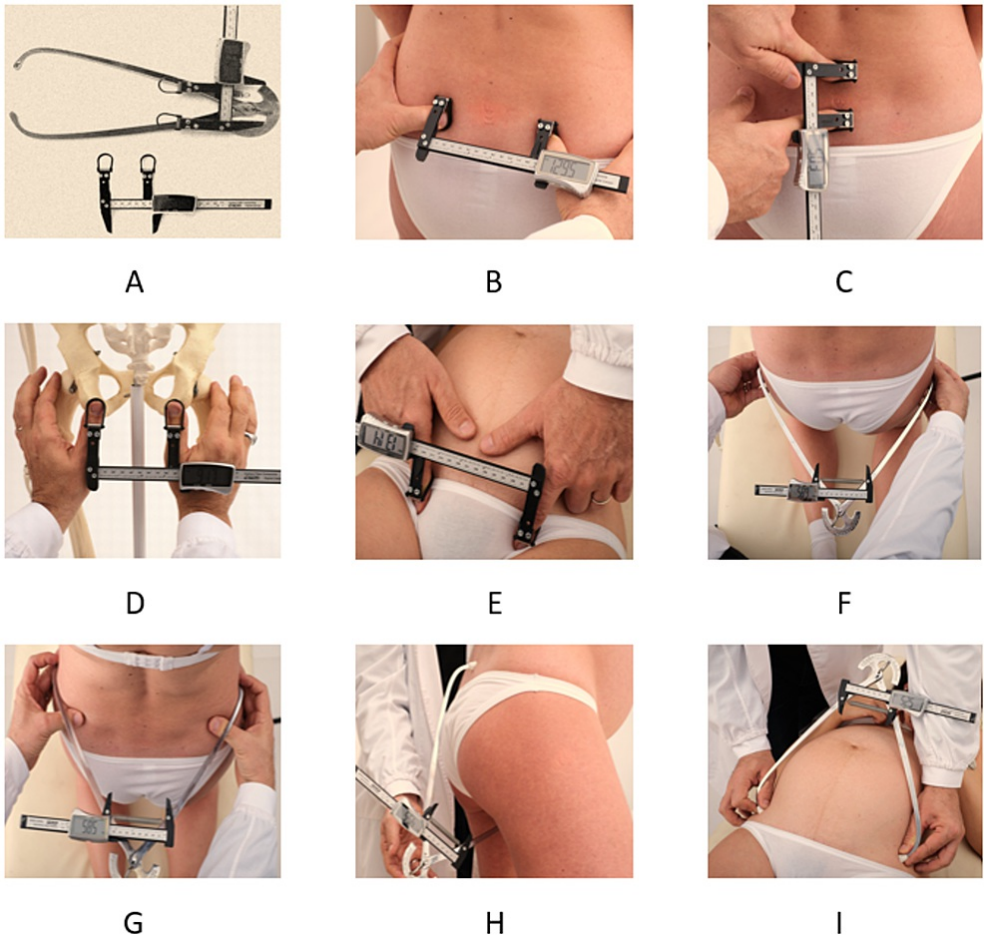
Instruments of dynamic external pelvimetry test and measurements of external pelvic diameters. A: The Digital Distance Indicator (DDI) (Metrica, Milan, Italy, EU, https://www.metrica.it/) and the prototype accessory Dynamic Pelvimeter. B-E: Small pelvic diameters. B: The transverse diameter of Michaelis’ sacral area. C: The longitudinal hemi-diameter of Michaelis’ sacral area. D: The inter-tuberous diameter. E: The base of the Trillat’s triangle. F-O: Large pelvic diameters. F: The bi-trochanteric diameter. G: The bi-cristal diameter. H: The external conjugate. I: The bi-spinous diameter.

The external pelvic diameters were measured in three different positions using the DEP test [16]. The measurement device used was the CE patented instrument Digital Distance Indicator (DDI) (Metrica, Milan, Italy, EU, https://www.metrica.it/) and its prototype accessory Dynamic Pelvimeter (DP) (Figure 1A). Both instruments allow the operator’s finger to maintain firm but delicate contact on the anatomical bone landmarks while the patient changes positions. Measurements were obtained after the subject had assumed each posture [16].

The smaller external pelvic diameters were estimated using the DDI. These included the transverse diameter of the sacral area of Michaelis (TD-SAM), the longitudinal hemi-diameter of the sacral rhombus of Michaelis, the inter-tuberous diameter, and the base of the Trillat’s triangle (BTT) (Figure 1B-1E). The larger external obstetric diameters estimated with the DP included the external anteroposterior conjugate, the bi-trochanteric diameter, the bi-cristal diameter, and the iliac bi-spinous diameter (Figure 1F-1I). The bony landmarks of each diameter were accurately individualized in each patient [15,16].

**Figure 2:**
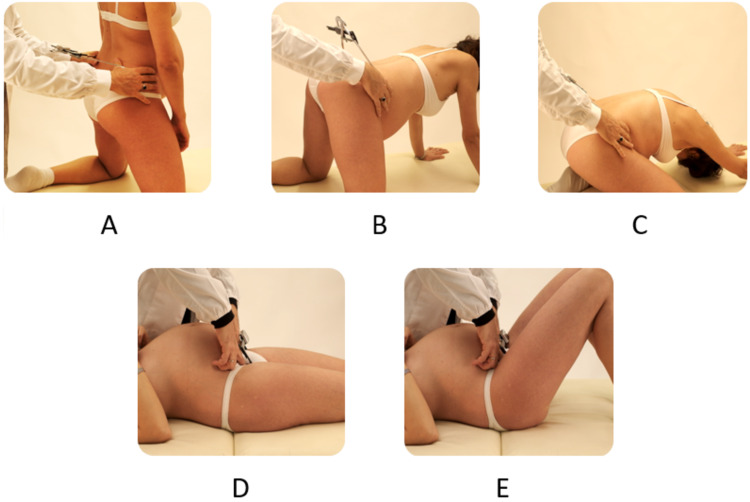
The different positions used for the dynamic external pelvimetry test. The maternal positions chosen for the dynamic external pelvimetry test were defined by different degrees of hip flexion. A-C: Kneeling positions. A: p1, straight-leg erect position. B: p2, hands-and-knees position. C: p3, kneeling squat position. D-E: Supine position. D: p1, straight-leg supine position. E: p2, flexed-leg position.

Diameters were estimated in the following three positions which involved varying degrees of hip flexion on the pelvis: p1, “kneeling erect” position (the erect straight-leg position) (Figure 2A); p2, “hands-and-knees” position (the bent-leg position, with 90° flexed hips, or “all fours” position) (Figure 2B); and p3, “kneeling squat” position (Figure 2C). The measurements of the anterior pelvic diameters (the BTT and the bi-spinous diameter) were the only ones obtained using the straight-leg supine position (p1) (Figure 2D), followed by supine with the lower limbs flexed to the maximum, keeping the feet in contact with the table, and the heels pulled in as close as possible to the ischial tuberosities (p2) (Figure 2E).

Participants were divided into the following two groups for statistical analysis according to the mode of labor and delivery: natural deliveries (the control group) and obstructed deliveries (the obstructed labor group). The natural delivery group included spontaneous labor in women who were free to change positions as preferred, no oxytocin required for induction or in the dilation phase of labor, and no medical assistance. The obstructed labor group included cesarean sections (unscheduled, after the onset of labor, all indications) and operative vaginal deliveries (pharmacological induction of labor and use of oxytocin during the dilation phase; deliveries assisted with either kiwi, forceps, or three or more Kristeller’s maneuvers; and 3° and 4° perineal lacerations). These births required medical assistance and were at an increased risk of potential peripartum and perinatal complications [17]. They were unsafe for mothers and newborns to manage at home or in a low-resource setting. Both groups included women with low-risk, term pregnancies with single fetuses in labor in our obstetrics ward.

The data derived from the DEP test were separated into groups: statistical analysis compared the obstructed labor group with the control group retrospectively. We recorded the dimension of each diameter measured in each position as well as the differences between diameter dimensions taken in each posture (p2-p1, p3-p1, p3-p2). The difference in diameters between postures refers to how the diameters changed on comparing measurements taken in the specified posture. The degree of change (DOC) corresponds to the elasticity of the pelvic tissues, allowing the pelvic spaces to adapt in shifting positions.

The OpenOffice spreadsheet (Apache OpenOffice 4.1.2. The Apache Software Foundation. Available at: http://www.openoffice.org) was used to record the measurements and obstetric outcomes. Pelvic diameter data are expressed in millimeters and reported as mean, standard deviation, minimum and maximum value, standard error of the mean (SEM), and 95% confidence intervals (95% CI). The measurement of normality with Kolmogorov-Smirnov test, unpaired t-test, chi-square test, receiver operator characteristics (ROC), area under the ROC curve (AUC θ-value), and the test for the minimum sample size required to calculate the ROC predictive value of the data were performed using the open-source statistical software StatsToDo (Statstodo Trading PTY LTD, Brisbane, Queensland, Australia; http://www.statstodo.com). A p-value of 0.05 and an AUC θ-value of 0.70 were considered significant.

## Results

The general characteristics of the study sample did not show significant statistical differences between groups (Table 1). Measurements of the external pelvic diameters in different positions and calculations of the difference between measurements obtained in the various maternal positions (a total of 2,770 values) were statistically analyzed in the groups. Similar to the previous study, the Kolmogorov-Smirnov test highlighted the normality of complete data distribution with a correlation between expected and actual cumulative frequency of >0.98 and a p-value of >0.1 [15].

**Table 1:** The general characteristics of study participants. Data are presented as mean and standard deviation (SD). Fetal weight was calculated using Johnson’s formula. The p-values from the unpaired t-test and chi-square test (*) are not statistically significant (N.S.).

	Obstructed labor group (11.4%)	Control group (88.6%)	P-value
	Mean (SD)	Mean (SD)	
Gestation (weeks)	35 (1.8)	34.6 (2.9)	N.S.
Age (years)	34.2 (2.5)	32.6 (4.7)	N.S.
Height (cm)	160.1 (6.4)	164.3 (6.1)	N.S.
Actual weight (kg)	62 (8.1)	69.5 (10.3)	N.S.
Pre-pregnancy weight (kg)	53.3 (4.9)	59.7 (10.6)	N.S.
Weight gain (kg)	8.7 (5.1)	9.8 (3.5)	N.S.
Symphysis-fundus height (cm)	36.1 (5.4)	33.8 (3.9)	N.S.
Fetal weight (g)	3,526 (716.6)	3,191.2 (595.8)	N.S.
Primiparas: n (rate)	6 (75%) *	46 (74.1%) *	N.S.*
Multiparas: n (rate)	2 (25%) *	16 (25.9%) *	N.S.*

The longitudinal hemi-diameter of the sacral area of ​​Michaelis (LHM-SAM), the BTT, and the distance between the ischial tuberosities showed significantly smaller anatomical rough measurements in all positions in the obstructed labor group compared to the control group (Table 2). The large external pelvic diameters measured using the DP were not statistically significant between the two groups in the positions studied (Table 3). The bi-trochanteric diameter and the external conjugate appeared more extensive in the obstructed labor group, while the DOC was more limited (Tables 4, 5).

**Table 2:** Measurements of small diameters in specified positions. T-test analysis between groups. Data are shown in millimeters as mean and standard deviation (SD). Values are the diameters measured in straight-leg (p1), legs flexed (p2), and kneeling squat (p3) positions. min: minimum value observed; max: maximum value observed; TD-SAM: transverse diameter of the sacral area of Michaelis; LHD-SAM: longitudinal hemi-diameter of the sacral area of Michaelis; LH-SAM: longitudinal diameter of the sacral area of Michaelis; Trillat’s base: the base of the Trillat’s triangle; N.S.: not statistically significant

Diameter/Position	Obstructed labor group		Control group		t-test
	Mean (SD)	Min-Max	Mean (SD)	Min-Max	P-value
TD-SAM/p1	129.8 (18.2)	92-150	122.8 (11.2)	97-148	N.S.
TD-SAM/p2	135.4 (18.6)	95-152	132.8 (11.7)	106-160	N.S.
TD-SAM/p3	137 (21.4)	90-154	133 (11.9)	104-161	N.S.
LHD-SAM/p1	39.1 (8.2)	32-53	47.3 (8)	22-72	0.008
LHD-SAM/p2	50.3 (7.1)	43-62	58 (10)	33-82	0.03
LHD-SAM/p3	55.1 (9.1)	45-72	62.9 (10.2)	44-90	0.04
LD-SAM/p1	108.8 (21.9)	90-150	108.6 (16.2)	62-156	N.S.
Inter-tuberous/p1	58.1 (11.4)	40-73	70.9 (12.5)	46-97	0.007
Inter-tuberous/p2	74.4 (19.6)	50-97	88.7 (12.1)	60-118	0.004
Inter-tuberous/p3	86.1 (17.7)	64-112	104.1 (13.1)	71-140	0.0008
Trillat’s base/p1	105.4 (17.4)	78-137	122.2 (13.1)	92-155	0.001
Trillat’s base/p2	97.7 (15.9)	74-126	111.7 (11.8)	82-143	0.003

**Table 3:** Measurements of large diameters in specified positions. T-test analysis between groups. Data are shown in millimeters as mean ad standard deviation (SD). Values are the measurements of diameters in straight-leg (p1), legs flexed (p2), and kneeling squat (p3) positions. Min: minimum value observed; max: maximum value observed; N.S.: not statistically significant

Diameter/Position	Obstructed labor group		Control group		t-test
	Mean (SD)	Min-Max	Mean (SD)	Min-Max	P-value
Bi-trochanteric/p1	350.6 (33.7)	320-414	339.6 (19.1)	302-376	N.S.
Bi-trochanteric/p2	342.1 (64.9)	328-427	351.1 (22.4)	307-396	N.S.
Bi-trochanteric/p3	375.1 (44)	332-449	371 (28.3)	286-430	N.S.
Bi-cristal/p1	283.3 (15.1)	258-300	283.4 (20.4)	220-329	N.S.
Bi-cristal/p2	280.6 (9.8)	273-299	285.8 (22.6)	225-329	N.S.
Bi-cristal/p3	280.8 (9.8)	270-292	278.7 (21.4)	221-320	N.S.
External conjugate/p1	239.5 (27)	206-277	226.7 (16.6)	194-271	N.S.
External conjugate/p2	239.5 (24.2)	215-275	228.1 (18.1)	191-286	N.S.
External conjugate/p3	241 (22.5)	218-280	232.2 (17.1)	201-284	N.S.
Iliac bi-spinous/p1	249.5 (24.6)	203-274	258 (17.3)	223-305	N.S.
Iliac bi-spinous/p2	244.7 (24.6)	203-269	253.2 (19.1)	217-302	N.S.

**Table 4:** Degree of change noted in the small diameters when comparing measurements in the specified positions. T-test analysis between groups. Data are shown in millimeters as mean and standard deviation (SD). Values reported are the difference in diameters measured between straight-leg and flexed-leg positions (p2-p1), straight-leg and kneeling squat positions (p3-p1), and flexed-leg and kneeling squat positions (p3-p2). Min: minimum value observed; max: maximum value observed; DOC: degree of change; TD-SAM: transverse diameter of the sacral area of Michaelis; LHD-SAM: longitudinal hemi-diameter of the sacral area of Michaelis; Trillat’s base: the base of the Trillat’s triangle; N.S.: not statistically significant

Diameter/DOC between positions	Obstructed labor group		Control group		t-test
	Mean (SD)	Min-Max	Mean (SD)	Min-Max	P-value
TD-SAM/p2-p1	5.5 (3.2)	3-10	10 (4.1)	4-21	0.004
TD-SAM/p3-p1	7.1 (6)	-2-15	10.1 (5)	1-21	N.S.
TD-SAM/p3-p2	1.5 (3.5)	-5-7	0.1 (3.1)	-10-8	N.S.
LHD-SAM/p2-p1	11.1 (3.5)	7-16	10.7 (4.9)	3-31	N.S.
LHD-SAM/p3-p1	16 (3.1)	12-19	15.6 (5.7)	6-31	N.S.
LHD-SAM/p3-p2	4.8 (4)	2-10	4.8 (4.1)	-3-17	N.S.
Inter-tuberous/p2-p1	16.2 (9.1)	2-26	17.6 (8)	4-39	N.S.
Inter-tuberous/p3-p1	28 (9.6)	16-46	33.2 (11.9)	12-65	N.S.
Inter-tuberous/p3	11.7 (6.7)	4-20	15.5 (7.1)	0-34	N.S.
Trillat’s base/p2-p1	-7.7 (3.1)	-11/-4	-10.5 (3.3)	-21/-5	N.S.

**Table 5:** Degree of change in large diameters when comparing measurements in the specified positions. T-test analysis between groups. Data are shown in millimeters as mean and standard deviation (SD). Values reported are the differences in measurements of each diameter between straight-leg and flexed-leg positions (p2-p1), between straight-leg and kneeling squat positions (p3-p1), and between flexed-leg and kneeling squat positions (p3-p2). Min: minimum value observed; max: maximum value observed; DOC: degree of change; N.S.: not statistically significant

Diameter/DOC between positions	Obstructed labor group		Control group		t-test
	Mean (SD)	Min-Max	Mean (SD)	Min-Max	P-value
Bi-trochanteric/p2-p1	8.1 (12.4)	-12-24	11.9 (7.6)	-1-32	N.S.
Bi-trochanteric/p3-p1	24.5 (22.4)	-8-43	31.3 (17.8)	-51-69	N.S.
Bi-trochanteric/p3-p2	16.3 (13.2)	0-31	19.3 (15.5)	-63-52	N.S.
Bi-cristal/p2-p1	-1 (11.5)	-22-17	2.4 (10.7)	-41-26	N.S.
Bi-cristal/p3-p1	-2.5 (11.1)	-19-14	-4.7 (10.3)	-36-20	N.S.
Bi-cristal/p3-p2	-1.5 (5.6)	-7-9	-7.1 (7.7)	-23-18	0.05
External conjugate/p2-p1	0.1 (5.6)	-7-9	1.3 (7.4)	-19-26	N.S.
External conjugate/p3-p1	1.5 (9.4)	-10-12	5.5 (8.6)	-12-41	N.S.
External conjugate/p3-p2	1.5 (6.5)	-8-11	4.1 (6.3)	-7-19	N.S.
Iliac bi-spinous/p2-p1	-4.8 (5.6)	-17-0	-5.8 (3.8)	-17-0	N.S.

The transverse diameter of Michaelis was narrower in the control group in the three positions, even if not statistically significant (Table 2). However, the difference in mobility between the p1 and p2 positions was substantial. The DOC of the transverse sacral diameter was more significant in the control group than in the obstructed labor group, showing a mean difference in diameter mobility between p1 and p2 positions of 4.5 mm (SEM = 1.5; 95% CI = 1.48-7.51; p = 0.004) (Table 4). The DOC (mobility) of the longitudinal hemi-diameter and the inter-tuberous diameter appeared almost identical between groups in all positions. The mobility of the BTT was different between the groups; mobility was limited in the obstructed labor group without reaching statistical significance (Table 4).

The mobility between the positions of the larger pelvic diameters did not show statistical significance between the extended leg position and the two bent leg positions (Table 5). Mobility of the bi-spinous diameter appeared within close range in the two groups. However, in the obstructed labor group, the bi-trochanteric, the external conjugate, and the bi-cristal diameter tended to have less mobility among all positions. The difference in the diameters between the iliac crests reached statistical significance in the transition from the “all-four” position to the kneeling squat position (mean = 5.6 mm; SEM = 2.8; 95% CI = -11.2-0.03; p = 0.05). The diameter decreased towards the squat position in the control group more than in the obstructed labor group (Table 5).

**Figure 3:**
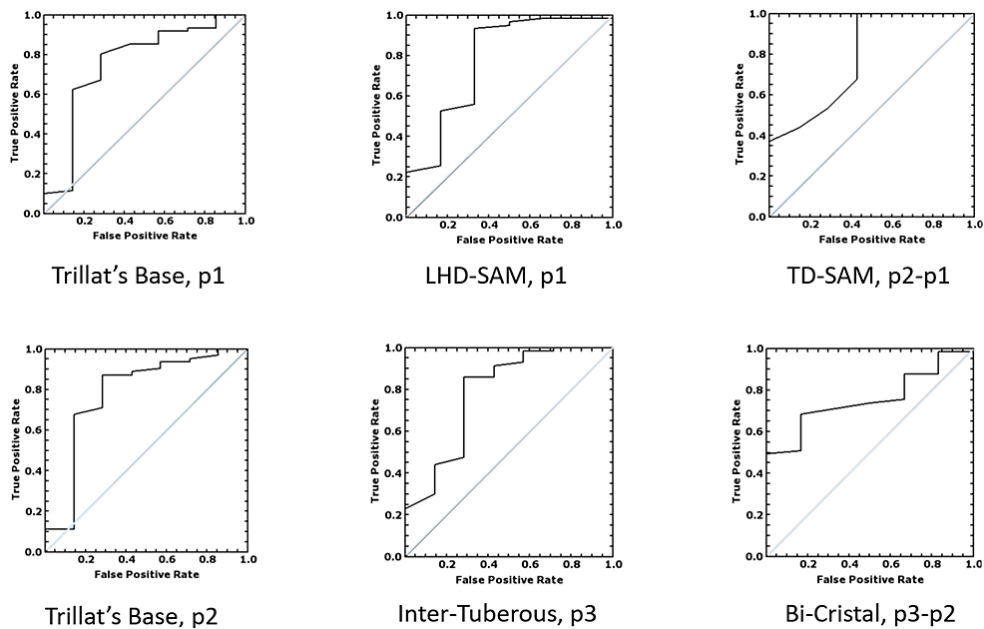
ROC curve plot of the most relevant diameters. Positions: straight leg (p1), flexed leg (p2), and kneeling squat (p3); the degree of change when the measurements taken in p2 and p1 are compared (p2-p1), as well as the measurements taken in p3 and p2 positions (p3-p2). TD-SAM: transverse diameter of the sacral area of Michaelis; LHD-SAM: longitudinal hemi-diameter of the sacral area of Michaelis; Trillat’s base: the base of the Trillat’s triangle

The ROC curve and the AUC calculated the predictive capacity of diameter measurements and mobility for natural birth/obstructed labor. The AUC θ-values of the statistically significant small diameters’ measurements and mobility and bi-cristal diameter’s mobility were calculated and are reported in Table 6. The most statistically significant measurement for each diameter was plotted (Figure 3). The diameter values that promised better screening accuracy were the following: the mobility of the TD-SAM between positions p1 and p2 (θ-value = 0.78; 95% CI = 0.57-0.99), the BTT measured in both positions (p1: θ-value = 0.80; 95% CI = 0.56 - 1; and p2: θ-value = 0.78; 95% CI = 0.54-1), and the inter-tuberous diameter measured in the kneeling squat position (θ-value = 0.78; 95% CI = 0.55-1) (Table 6, Figure 3).

**Table 6:** Area under the ROC curve analysis of statistically significant diameters. The significant predictive capacity analysis is for AUC θ > 0.70, representing the degree of separability: a θ-value above 0.50 refuses the null hypothesis to distinguish values between the two groups. Positions: straight-leg position (p1), flexed-leg position (p2), and kneeling squat position (p3); the difference in the diameter when measured in the straight-leg and flexed-leg positions (p2-p1); the difference in the diameter when measured in flexed-leg and kneeling squat positions (p3-p2). ROC: receiver operator characteristic; AUC: area under the ROC curve; SEM: standard error of the mean; 95% CI: 95% confidence interval of the θ-value; TD-SAM: transverse diameter of the sacral area of Michaelis; LHD-SAM: longitudinal hemi-diameter of the sacral area of Michaelis; Trillat’s base: the base of the Trillat’s triangle

Test (diameter/position)	AUC (θ)	SEM	95% CI
LHD-SAM/p1	0.7759	0.1307	0.521-1
LHD-SAM/p2	0.7428	0.1053	0.5375-0.9481
LHD-SAM/p3	0.7198	0.1206	0.4846-0.955
Inter-tuberous/p1	0.7794	0.0823	0.6189-0.94
Inter-tuberous/p2	0.7043	0.1229	0.4647-0.9439
Inter-tuberous/p3	0.7832	0.1153	0.5584-1
Trillat’s base/p1	0.803	0.1196	0.5698-1
Trillat’s base/p2	0.7811	0.12	0.5471-1
TD-SAM/p2-p1	0.7846	0.1068	0.5764-0.9927
Bi-cristal/p3-p2	0.7544	0.0796	0.5991-0.9097

The post hoc test was performed to calculate the minimum sample size required to obtain a significant result within our range (θ-value = 0.7-0.8; SEM = 0.08-0.12). The test range required a total sample size of 16-44 participants (8-22 per group) to have a significant ROC analysis. The present study involved a total of 70 pregnant women.

## Discussion

This pilot study is the first to examine the diameters of maternal external pelvimetry in different positions and correlate with delivery outcomes. It lays the foundation for more extensive and in-depth multicenter clinical trials. Previous studies evaluated the rough anthropometric data from external pelvic diameters to predict obstetric outcomes [10-14]. These studies evaluated the transverse and longitudinal diameter of the Michaelis sacral area and some outer diameters of the upright maternal pelvis. The TD-SAM was reported to be the most accurate test for identifying the risk of pelvic contraction; moreover, the longitudinal diameter of Michaelis’ rhombus and maternal height also had an excellent predictive index [11-14].

In this pilot study including a limited number of patients, maternal height, symphysis-fundus length, and neonatal weight did not reach statistical significance. However, there was an expected difference between groups.

The measurements of the transverse diameter of the Michaelis area in different positions were similar between groups. The LHD-SAM measurements in the three postures were associated with the type of delivery. However, The LD-SAM was not associated with obstructed labor, in agreement with our previous study on a large group of pregnant women [18]. The longitudinal hemi-diameter distance refers to the anatomical shape of the sacral rhombus of Michaelis and the angle of the sacral promontory, which, by projecting forward, influences the space of the posterior pelvic inlet. However, the data do not support the influence of the mobility of the lumbosacral junction on fetal descent [5,6]. The mobility of the TD-SAM and the bi-cristal diameter were broader in the control group. The inter-tuberous diameter, not mobility, was smaller in the obstructed labor group than in the control group, as proven by clinical experience and previous literature [17]. The measurement of the inter-tuberous diameter provides the transverse width of the space between the bones and the soft tissues of the pelvic outlet. Ischial tuberosities are in an anatomical relationship with the subpubic angle through the ischiopubic rami and are associated with the transverse midpelvic space between the ischial spines [17].

Similarly, the measurement and not the mobility of the base of the Trillat’s triangle (distance between the two inguinal creases measured on the upper edge of the pubis) is associated with obstetric outcomes (Tables 2, 4). It corresponds to the transverse width of the midpelvis, as reported by Trillat in the original publication [19]. The BTT is influenced by the shape of the pubic branches, the pubic symphysis, and the subpubic arch angle (SPAA).

Our previous study highlighted the linear correlation between the LHD-SAM and the BTT [15]. Linear correlation was recorded between the LHD-SAM measurement and the amplitude of the SPAA measured by two-dimensional ultrasound in the “flexed legs” p2 position [15,20]. Magnetic resonance studies have demonstrated the correlation between the SPAA, the internal conjugate, and the transverse diameter of the midpelvis, highlighting an anatomical harmony between the dimensions of the birth bone canal [21,22]. The pelvis appears as an in-section composed structure functioning as a dynamic whole and adapting to different human biological functions. DEP aspires to be a practical clinical test for evaluating pelvic mobility [15,16]. The DEP test of the small diameters of the maternal pelvis requires direct contact of the operator’s fingers on the bony reference landmarks during the change in positions. The bony landmarks must be accurately individualized in each patient. Good inter-operator and intra-operator agreements have been reported for the test [15].

The DEP test performed on the longitudinal diameters and hemi-diameter of the SAM resembles a modified Schober’s test, which analyzes the flexion capacity of the lumbosacral spine [15]. The LHD-SAM is more easily measured using the DDI than the longitudinal diameter and more closely reflects the state and mobility of the sacral promontory and the lumbosacral junction [15,16]. LD-SAM and LHD-SAM correlate linearly in the three positions studied (unpublished personal data from a preliminary study). Finger contact on short-distance bone landmarks is easier to maintain during a patient’s movements for shifting positions [15].

**Figure 4:**
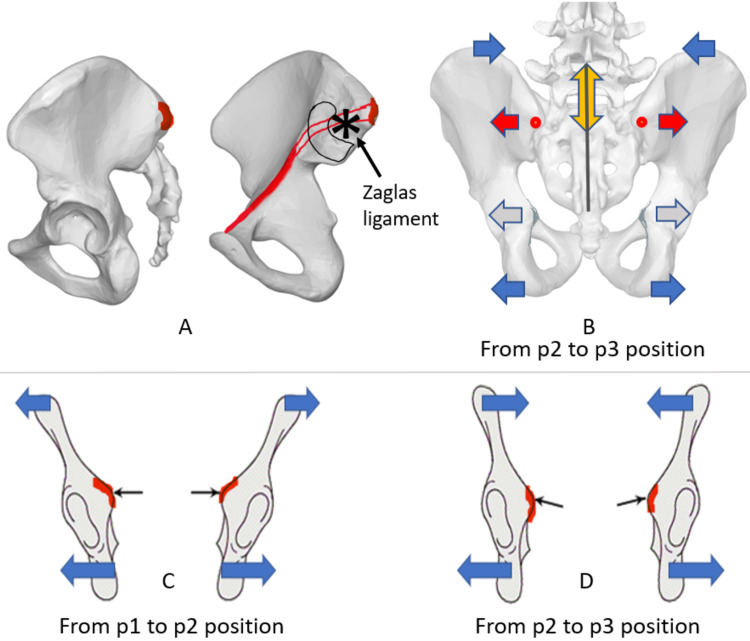
Zaglas ligament, pelvic mobility, and the inclination (angle) of the pelvic brim. The inclination of the pelvic inlet bone edge changes with maternal postures. Moving into the squat position enlarges inferior diameters and narrows the superior diameter. The change of the inclination of the pelvic brim appears to favor fetal descent. Moreover, the region of the transverse diameter of Michaelis is anatomically related to the pelvic inlet. The inter-tuberous diameter of the outlet is functionally associated with the transverse diameter between the ischial spines in the midpelvis [17]. Zaglas ligament is one of the bands of the posterior sacroiliac ligament [23]. A: Lateral and medial view of the ilia. B: Posterior view of the pelvis. C, D: A simplified figure of the left and right ilioischial complexes of the pelvis (pubis and sacrum are removed) passing perpendicularly through the innominate line of the pelvic brim. p1: kneeling erect position; p2: all-fours position; p3: kneeling squat position. Asterisk: reported attachment site of the Zaglas ligament on the medial ileum; red line: pelvic brim; red dots: posterior superior iliac spines; red arrows: mobility of the transverse diameter of the Michaelis area; yellow double-headed arrow: mobility of the longitudinal hemi-diameter of the Michaelis area; gray line: the longitudinal diameter of the Michaelis area; blue arrows: mobility of the bi-cristal and inter-tuberous diameter; light gray arrows: inter-spinous diameter of the midpelvis; small black arrows pointing to red marks in C and D panels: together, they indicate the pelvic brim slope in each position.

The lower landmark of the LD-SAM is located at the upper margin of the gluteal cleft, close to the sacrococcygeal junction, where the skin and the underlying fascia slide widely on the periosteal surface. The LHD-SAM has the same uppermost mobile landmark, the tip of the spinous process of the fifth lumbar vertebra, as the longitudinal diameter. The lower extremity of the LHD-SAM is relatively immobile between the two posterior superior iliac spines (PSIS) on the second sacral segment. There, the so-called Zaglas ligament is reported to be localized. It is one of the deep fascial structures posterior to the sacroiliac joint that accomplishes the axis of motion of the sacrum between the ilia (Figures 4A, 4B) [23]. The LHD-SAM measurement was the lowest in the obstructed labor group, while the mobility did not differ between groups.

The extremities of the transverse diameter of the sacral area of Michaelis are the prominences of the PSIS, which are located on the same anatomical plane as the ilia innominate line (Figure 4A) [16]. In this pilot study, the anatomical mobility of the pelvic tissues measured between the PSIS was strongly decreased in the obstructed labor group compared to the control group. By changing the maternal position from standing to “all-fours,” the layers of the thoracolumbar fascia, the lumbar-ileo-sacral ligaments, and the sacroiliac joint and capsule slide over each other to allow the counternutation of the sacral base. It appears together with the flexion of the lumbosacral junction (LHD-SAM lengthening) as “the back is opening” to give birth, as reported by traditional midwives [9,10]. The rearward movement of the upper sacrum is confirmed by the tendency of the external conjugate to increase in size between the upper edge of the pubic symphysis and the first sacral segment from the p1 position to the squat position to a greater extent for the control group (Table 5) [15]. When measurements are compared with each maternal position change, the amplitude and mobility of the Michaelis transverse diameter are linearly related to the amplitude of the bi-cristal diameter; the TD-MSA also correlates with the mobility of the external conjugate in all positions [15].

The mobility of the transverse Michaelis diameter, the width of the Trillat’s triangle base, and the inter-tuberous diameter can be used to best screen for obstructed labor. This small sample does not allow for a more complete and in-depth analysis. Nevertheless, an ROC analysis on a population of 451 third-trimester pregnant women indicated that the TD-MSA dynamic test in changing positions, considering the cut-off at 30th percentile, has a sensitivity of 0.86, a specificity of 0.87, a true-positive rate of 0.80, a true-negative rate of 0.98, an accuracy of 0.95, a false-positive rate of 0.01, and relative risk of 6.98 with 95% CI of 5.1-9.4 (chi-square test; p < 0.0001) [18].

The difference in amplitude between maternal positions of the bi-cristal diameter was statistically different between the two groups studied. Specifically, when we compared the measurements taken in the standing p1 position to those obtained in the “hands-and-knees” p2 position, the diameter decreased in the obstructed labor group, while it increased in the control group, in agreement with our previous results (Table 5) [15]. However, in the squat position, the bi-cristal diameter decreased more in the control group than in the obstructed labor group.

As reported in the previous study, the maximum amplitude of the bi-cristal space is measured in the middle position (the “all-fours” position). The diameter becomes narrow in the squat position and narrower still in p3 than the measurements obtained in the p1 position [5,15]. The distance between the iliac crests measured at the greatest width is the upper limit of the bony pelvis; the distance between the ischial tuberosities (pelvic outlet) is its lower limit (Figures 4B-4D). The dynamic behavior of the inter-tuberous diameter mirrors the mobility of the TD-SAM and LHD-SAM (Table 2). Diameters steadily increase from p1 to p3, as previously reported for most pelvic diameters using magnetic resonance and external pelvimetry [4-6,15]. The narrowing of the pelvis upper extremity and the opening of the pelvic outlet would likely change the inclination of the bone ridge of the pelvic inlet innominate line (Figures 4B-4D). The flexion of the lumbosacral junction, indirectly highlighted by the mobility of the LHD-MSA, helps open the inlet space at the level of the angle of the sacral promontory [5-7,16].

This study follows the obstetric literature that considers the static cephalopelvic ratio (the comparison between the anatomical measurements of the maternal pelvis and the malleable diameters of the fetal head) to be of fundamental importance for optimal delivery. Nevertheless, the results presented here, showing external pelvic diameter rigidity and narrowing in the obstructed labor group, indicate that the mobility and adaptability of the pelvic diameters of the birth canal in different maternal positions is an additional aspect to consider in assessing the capacity and possibility of physiological birth. The size of the diameters that change with maternal positions and the mobility of the pelvic joints define the space for easy progression of the fetal head. The mobility of external diameters is the DOC when different positions are assumed. The DOC of the transverse diagonal of the Michaelis’ sacral rhombus and the distance between the iliac crests correlate with the obstetric outcomes (Tables 4, 5) [15].

The measurement of the external diameters is not simply the distance between two bony landmarks, as is the case for internal radiological pelvimetry. Nevertheless, the condition of soft tissues gliding upon the pelvic bone, which helps define the space available for the descent of the fetal presenting part, must be considered as well. As assessed by ultrasonography, soft tissue biomechanics and adequate mobility of the pelvic viscera are associated with the possibility of vaginal birth [24,25].

The elasticity and plasticity of the connective tissues, in general, and pelvic connective tissue, in particular, change as pregnancy progress, leading to greater segmental mobility to prepare the maternal-fetal unit for the key life birth event. The pelvis is a functional composite of bones, fascia, ligaments, muscles, and viscera that interact to perform physiological functions and adjust during pregnancy, labor, and delivery. Studies have shown an enlargement of the SPAA, an increase of the elbow joint extension, and gain of the mobility of the pelvic viscera during pregnancy [24,25]. The mobility of the pelvic viscera and joints is an indirect measure of the biomechanics of the connective tissue, influenced by placental hormones. Dietz et al. highlighted the lower percentage of damage to the supporting tissues of the pelvic floor due to childbirth in pregnant women with greater mobility of the pelvic organs and bone joints. They ascribed tissue stiffness and hypomobility as a cause for the increase in the rate of operative deliveries. Tissue rigidity during prolonged labor causes damage to the pelvic floor and supporting fascia, leading to symptoms of pelvic floor dysfunction [24,25].

Similarly, radiological examinations have found damage to the insertions of the levator ani muscle and pelvic bone disruptions in women after prolonged labor and dystocia [26]. Moreover, lower back pain and pelvic girdle symptoms before and during pregnancy manifest the altered biomechanical properties of pelvic tissues; pain and tissue mobility are inversely related. Patients reporting higher pain ratings during the third trimester of pregnancy are at risk of cesarean section, assisted delivery, and a longer duration of labor [27].

The diameters of the external pelvimetry are anatomically and functionally influenced by pelvic fasciae and joints [5,6,8,28,29]. The mobility of the sacroiliac joints, the pubic symphysis, the thoracolumbar fascia, and its ligamentous thickenings modulate the mobility of the entire maternal pelvis and the malleability of the birth canal. The deepest part of the posterior layer of the thoracolumbar fascia inserts into the periosteum of the sacrum and maintains continuity with the sacrotuberous ligaments and along the ischiopubic falciform ligament until it reaches the subpubic arch ligament and superiorly until it reaches the base of the skull [28]. The anterior layer of the thoracolumbar fascia involves the crura of the diaphragm, the psoas, and the quadratus lumborum muscles [28]. It has a central relationship with the retroperitoneal renal fascia and genitourinary organs. The thoracolumbar fascia from the composite sacral area continues inside the pelvis through the iliolumbar ligaments, the anterior sacroiliac joint capsule, and the parietal endopelvic fascia supporting ligaments of the pelvic viscera are inserted [28,29].

The DEP test can reveal the mobility of the fascial layers permitting the transmission of forces during biomechanical body functions. Childbirth is considered to be a biomechanical process as bipedal locomotion and is similarly subjected to the selective pressure of evolution [5,6,30]. Stiffness and hypomobility prevent the elastic deformation of a hydrated fascial system from transmitting the kinetic forces and saving metabolic energy [28]. Moreover, the posture imposed by bipedal locomotion in humans produces a gravitational pull on the pelvic structures. Selective pressure widens the transverse pelvic diameters, minimizing the effects of gravitational strain on the pelvic floor and viscera [30]. Moreover, it changes the suspensory ligaments of pelvic organs; inter-visceral mobility is permitted by insertional fasciae, and peri-visceral fat and loose connective tissue help dissipate the effects of gravitational force [28]. The visceral fascia is a connective tissue layer that envelops the subperitoneal portion of the pelvic organs, attaching them to the parietal fascia. It consists of collagen, muscle fibers, nerves, and blood vessels. The pubocervical and rectovaginal fasciae are divisions of the visceral pelvic fascia as uterosacral and cardinal ligaments. The parametrium, the paracervix, the lateral ligament of the rectum, and the presacral fascia are dense connective tissues surrounding the viscera and lying between the parietal and visceral pelvic fascia; supporting the vascular and neural structures of the pelvis and allowing for physiological volume variations of the pelvic viscera and freedom of movement between viscera are among their key functions [24,25,28,29].

Peri-visceral fasciae and visceral ligaments are inserted on pelvic parietal fasciae surrounding the bone, muscle, and joint structures, linking the internal to the external body structures. Childbirth and bipedalism are different functions involving the same pelvic anatomical structures with different biomechanics, suitable for each role. Such a process requires enduring regulating selection and evolution that are beginning to be studied and elucidated [30]. Adequate mobility of the various tissue layers at different magnitudes is crucial for static and dynamic functions of the entire human physiology. Locomotion, thermoregulation, nutrition, reproduction, and childbirth are closely related in their never-ending evolutionary history, and only heritable traits evolve by natural selection. Several studies have indicated substantial heritability for pelvic and fetal dimensions, as reported by Pavličev et al. [30]. Pelvic rigidity/CPD implies that the pelvis is contracted, leading to prolonged/obstructed labor and maternal-fetal morbidity and mortality [30]. Operative delivery and cesarean section are performed to avoid fetal injury and pelvic floor damage. However, women born through cesarean section are more likely to experience CPD and cesarean delivery [30]. The extensive possibility to perform cesarean sections for CPD indication has increased rates of CPD, influenced pelvic architecture, and affected human anatomy and metabolism over decades and not within thousands of years. Multigenerational studies have established evidence for the inheritance of CPD via the maternal genome [30]. Hence, improving and encouraging natural childbirth has become imperative for modern medicine in defending transgenerational human health.

The data described in this study cannot be compared with the scarcely available literature. The average maternal anthropometric and pelvimetric data vary widely due to the differing ethnicity, methods of detection, and measurement tools [10-14]. However, according to previous studies, measurements of the Michaelis sacral rhombus diameters serve as predictors of CPD and dystocia.

This study has the following limitations: the pilot study was single-center research, the sample size of the obstructed group, the different sizes of the groups, and an extensive SD of some diameters and differences between positions [15]. However, its purpose was to analyze the results and literature and give operative indications for multicenter clinical trials on a broader population to elucidate the possible relationships between pelvic mobility and obstetrics outcomes. Further research must evaluate the adjuvant role of manual therapies and exercise on pelvic hypomobility.

## Conclusions

Childbirth is an evolutionary and dynamic process affected by the form and function of pelvic tissues and human behaviors. The size and mobility of pelvic diameters influence obstetrics outcomes. The DEP test is a low-tech clinical assessment suitable for use in any socio-economic context. It may help obstetricians screen for obstructed labor and prevent maternal-neonatal morbidity that dystocia and CPD imply. Large clinical trials are needed to bridge the knowledge gap on the biomechanics of human birth and to determine the screening value of the pelvic mobility test.
